# Danish Linguistic Validation and Cultural Adaptation of the LIMB-Q Kids

**DOI:** 10.3390/children10071107

**Published:** 2023-06-25

**Authors:** Christopher Emil Jønsson, Lotte Poulsen, Jan Duedal Rölfing, Harpreet Chhina, Anthony Cooper, Jens Ahm Sørensen

**Affiliations:** 1 Research Unit for Plastic Surgery, Odense University Hospital, 5000 Odense, Denmarkjens.sorensen@rsyd.dk (J.A.S.); 2 Department of Clinical Research, University of Southern Denmark, 5230 Odense, Denmark; 3 Children’s Orthopaedics and Reconstruction, Aarhus University Hospital, 8200 Aarhus, Denmark; 4 Department of Orthopaedics, Faculty of Medicine, University of British Columbia, Vancouver, BC V5Z 1M9, Canada; 5 Department of Orthopaedics, BC Children’s Hospital, Vancouver, BC V5Z 4H4, Canada

**Keywords:** patient reported outcome measure, PROMs, health-related quality of life, HRQL, translation and cultural adaption, TCA, lower limb deformities, pediatrics

## Abstract

(1) Background: Lower Limb deformities include many conditions where pain and physical limitations negatively impact the health-related quality of life (HRQL) of children. The ideal way to assess this impact is by using a Patient Reported Outcome Measure (PROM). Such a condition-specific PROM is under development, called LIMB-Q Kids. This study aimed to perform a translation and cultural adaption (TCA) of the LIMB-Q Kids for use in Danish-speaking children. (2) Methods: TCA guidelines established by the World Health Organization (WHO) and the Professional Society for Health Economics and Outcomes Research (ISPOR) were followed. This process consisted of two independent forward translations, a reconciliation meeting, a backward translation with an assessment, an expert meeting, cognitive interviews with patients, editing based on the interviews, and proofreading. (3) Results: The TCA process contributed to the Danish version of LIMB-Q Kids. The reconciliation meeting resulted in a reconciled Danish version. The revision of the backward translation led to 16 corrections, and after the expert panel meeting, 26 changes were made. Twelve cognitive interviews led to nine changes, which were validated by two further interviews. Proofreading led to no further comments. (4) Conclusions: The TCA process led to a linguistically validated and culturally adapted Danish version of LIMB-Q Kids. This version is being used in the international field test study.

## 1. Introduction

Pain and physical limitations negatively affect the health-related quality of life (HRQL) of children living with a lower limb deformity [1]. Moreover, the appearance of a limb with deformity and physical impairment often has a psychological impact on the child, affecting their social activities and relations [2].

Children can suffer from a wide range of deformities, such as lower limb deficiency and rotational and angular deformities of the hips, knees, ankles, and feet [3]. Further, different conditions can arise from a multitude of congenital, acquired, and idiopathic etiologies [4]. A common lower limb deformity is Leg Length Discrepancy (LLD), where one leg is longer than the other. It is debated as to which degree small length differences affect the walking function, but studies show that even small discrepancies correlate with increased odds for hip and knee pathology [5]. LLD can be corrected with different treatment options, spanning from conservative approaches such as a shoe lift to invasive procedures such as epiphysiodesis or limb lengthening [6]. In the same way, a limb-threatening condition can, in some cases, be managed by limb salvage surgery and, in others, by an amputation. Both approaches impact the patient physically and psychologically; hence their overall HRQL is impacted [7].

When planning or evaluating such complex treatments, it is essential to not only focus on objective measures such as radiographs, but to a great extent, include the patient’s perspective to provide patient-centered care. A Patient Reported Outcome Measure (PROM) is a standardized tool for making evaluations of functional health status (FHS) and HRQL. The questions in the PROM can uncover aspects of the patient’s life that are affected by the deformity, and thereby determine how different treatments influence a patient’s life [8]. To obtain reproducible and dependable data, it is essential to use a valid PROM developed with standardized methods [9,10].

After performing a systematic review, Chhina et al. concluded that no valid PROM currently exists for this patient group, which ultimately led to the development of the LIMB-Q Kids [11]. LIMB-Q Kids is designed specifically for use in children with lower limb deformities to measure their HRQL [2]. The development of LIMB-Q Kids involved an international qualitative study in determining what matters most to children with lower limb deformities [12].

LIMB-Q Kids is based on a conceptual framework developed through 79 cognitive interviews conducted in Canada, Ethiopia, India, and the USA with children who had a wide variety of lower limb deformities and their parents [2,12]. The content validity was then established by 40 cognitive interviews with patients, parents, and experts, where they reviewed a preliminary version of LIMB-Q Kids [2]. As a result, LIMB-Q Kids now consists of 159 items and 11 scales, which each measure a concept of interest for this patient population [2]. Despite the extent of more than 150 items, the cognitive interviews found a high degree of coherence to the questionnaire for the participants. The next step in the development process is the international field test, where data collected from many sites will be used for the Rasch analysis to perform item reduction and psychometric validation [2]. The cross-continental development ensures a high general, international validity, but in order for the PROM to be used in another country, it is necessary to conceptually translate it into the country’s native language and to ensure cultural adaption [13].

The aim of the present study was thus to translate and culturally adapt LIMB-Q Kids to Danish.

## 2. Materials and Methods

This study was reported to the Danish Data Protection Agency under the collective permission held by the Region of Southern Denmark prior to its beginning, Journal nr.: 21/41544. There is no need for approval from the Regional Scientific Ethical Committee of Southern Denmark as this study is a questionnaire and interview survey, which by Danish law, does not need ethics approval.

The participant’s parents or legal guardians have given their verbal and written informed consent to participate in this study. Further, all participants have given their verbal assent to participate. All children over the age of 15 have also given their written consent.

**Figure 1:**
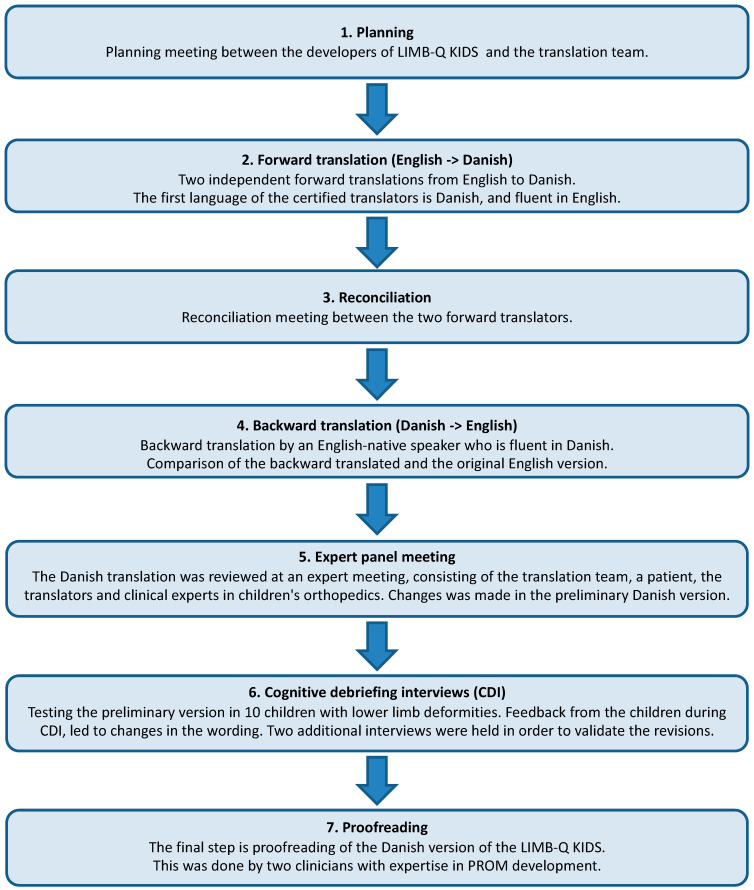
Illustration of the translation and cultural adaption (TCA) process.

The rigorous translation and cultural adaptation (TCA) guidelines established by the World Health Organization (WHO) and the Professional Society for Health Economics and Outcomes Research (ISPOR) were applied [13]. This approach ensured the validity and comparability of the Danish version of LIMB-Q Kids internationally, and consisted of the following seven steps (Figure 1):

1. Planning

A meeting between the developers of LIMB-Q Kids (H.C, A.C) and the translation team was held, where the steps for translation were planned. This study obtained approval from the Region of Southern Denmark prior to its beginning;

2. Forward translation

A professional translator and the first author (C.E.J) each performed an independent forward translation. In line with the TCA guidelines, both translators are fluent in English and have Danish as their first language. The qualifications of the professional translators were ensured by using only a certified translator with a master’s degree in the given language. The translators were instructed on the importance of maintaining the conceptual meaning of the items while keeping them as easily understandable and translatable as possible. Special attention was paid to keeping the reading level as low as possible, considering our target patient population of children between 8 and 18 years;

3. Reconciliation

During a reconciliation meeting, the two independent forward translators compared their translations, resulting in a harmonized Danish version;

4. Back translation

A backward translation was then made by a professional translator who is fluent in Danish and has English as a first language. This new English version was then compared with the original version by the developer of LIMB-Q Kids (H.C), and the discrepancies were discussed with the translation team, consisting of C.E.J., L.P., J.D.R., and H.C. The translation issues were discussed by mail, until all concurred on a suitable solution. For specific translational details, please refer to the “results” section below. This resulted in the second Danish version;

5. Expert panel meeting

During the expert panel meeting, the clinical relevance of the items was ensured. The expert meeting panel included the developer of LIMB-Q Kids, a patient with a lower limb deformity and the patient’s father, three orthopedic surgeons, a specialist nurse, and a physiotherapist, all with expertise in treating children with lower limb deformities. Finally, we also included an expert in PROM development (L.P.), two professional translators, as well as the first author (C.E.J.). All participants were given the preliminary Danish version of LIMB-Q Kids and were instructed to look at it thoroughly prior to the meeting. This meeting resulted in a third Danish version;

6. Cognitive debriefing interviews (CDI)

CDIs were then conducted by the first author (C.E.J.). Patient recruitment was conducted by the Department of Orthopaedics at Aarhus University Hospital, with the patient inclusion criteria being children between the ages of 8 and 18 years old, diagnosed with a lower limb deformity, and with Danish as their first language. Exclusion criteria were children with comorbidities that could affect their HRQL, cognitive disorders which affect the ability to speak and read, as well as isolated hip and foot defects. Selective sampling was used to ensure a heterogeneous patient population for this study.

All CDIs were conducted by video conference due to the COVID-19 pandemic, with the attendance of the participant and their parents. CDIs focused on the comprehensibility and understanding of the instructions, response options, and items. This was conducted according to the interview guide provided by the Q-portfolio team [14]. For every instruction, response option, or item, the participants were guided by the interviewer to describe, in their own words, what was asked of them [15]. By using screen sharing, the interviewer was able to highlight the items as the participant was evaluating them. When the children were asked how they understood the items, an effort was made to notice if they had an increased reflection time, as it could be due to comprehension problems. At the end of the CDIs the participants and the parents were also asked if they had any comments to the content of the LIMB-Q Kids and the translation.

As the first round of CDIs (10 interviews) resulted in changes, an additional round of CDIs (2 interviews) was conducted with the intention of evaluating whether the misconceptions had been clarified;

7. Proofreading

Finally, the Danish version of the PROM was proofread by two clinicians to generate the final version of the Danish LIMB-Q Kids.

## 3. Results

A complete list of corrections made during the TCA process can be seen in Table S1: “Overview of the translation process with Danish explanations.”

### 3.1. Forward Translation

The first author (C.E.J) and the professional translator each made an independent translation of the LIMB-Q Kids. At the reconciliation meeting, thirty-three instructions, response options, or items were found to be incongruent. The translation of the word “shaky” was discussed, as its literal Danish translation does not accommodate the same connotations of instability that is in the original English item. A Danish word was added as a supplement to emphasize these connotations of instability. The incongruences in item translations were primarily caused by the use of different synonyms or the translators making equivalent adaptations of the conceptual meaning of an item. In such cases, the most comprehensible translation with the lowest reading level was favored. The majority of the items in the English version of LIMB-Q Kids are written at or below a grade three reading level suitable for 8-year-old patients. This resulted in version one of the Danish translation.

### 3.2. Backward Translation

The comparison between the backward translation of Danish version one and the original English version resulted in 16 changes. Nine of these sixteen corrections were the same minor change made in a reoccurring instruction (See Table S1). In addition, identical changes were made to three items where the Danish translation was corrected from a phrase meaning “standing up” to a word meaning “standing” as in the original to ensure the meaning was retained. Another essential part of the comparison of the backward translation was to ensure the low reading level of the original LIMB-Q Kids. This step resulted in a second Danish version.

### 3.3. Expert Panel Meeting

The expert panel concurred on 26 minor alterations in item/instruction/response options. These changes were made to ensure the items reflect the words commonly used by the patients and the healthcare professionals, and to keep the items as easily understandable as possible. The meeting was held online in Danish, with English questions for the LIMB-Q Kids developer.

Some identical changes were applicable to several items, such as four items concerning whether the patients would like to be able to walk or run faster. The expert panel questioned that the original translation of the items does not accurately convey the focus of the items, which should be on the patients’ essential desire to walk or run faster. The items were revised to reflect the meaning of the original items. The former adaption was closer to the English word “want” as the chosen substitute comes closer to “would like to” as used in the English item (See Table S1).

Additionally, five items that used the word “shaky” were discussed, as their literal Danish translation was not found to describe the same degree of instability as present in the original item. To address this issue, two Danish synonyms for instability were added in brackets to be tested in the next CDIs. Additionally, the combination “swollen or puffy” is used four times in LIMB-Q Kids, and it was decided by the expert panel that the Danish word for puffy was redundant in the Danish translation, and it was therefore removed from the items.

In the expert meeting, two items involving the translation of “Dislike” were discussed, as its Danish translation comes closer to the term “Do not like,” which, in combination with the response option “Never,” results in a double negation. A more accurate translation could not be found.

All changes were assessed by the participating patient, and the consensus among the experts was to further evaluate the changes in the CDIs, and thus the third version was ready in Danish for testing in the CDIs.

### 3.4. Cognitive Debriefing Interviews

Initially, 10 CDIs were conducted with a diverse patient group that fitted the inclusion criteria for this study. The biological sex, age, type of lower limb deformity, and treatment status of the included participants at the time of the interview are presented in the demographics table (See Table 1).

The CDIs did not display any major comprehension problems in the third Danish translation of the LIMB-Q Kids, and the participants found the items relevant, easily understandable, and relatable to their own experience of having a lower limb deformity.

Four minor issues were identified in the CDIs that, when accessed by the translation team and the international developer, led to a revision. The first was in items involving “sitting cross-legged,” which is often described as a literal translation of the expression “Tailor sitting position” in Danish. This expression was used in the third Danish translation, combined with the literal translation of “sitting cross-legged” in a bracket. Some of the younger children were confused as they did not know this expression. It was therefore decided to add the expression in brackets instead of the literal translation to ensure the understandability of the item (See Table S1).

Secondly, corrections were made in two items that focused on walking on a bumpy road. The interviews showed that three of the younger participants did not understand the Danish word for “bumpy”. It was therefore decided to add a Danish literal translation of “holes in the road” in a bracket.

The third correction was in two items involving the translation of “Dislike” that creates a double negation, which was evaluated in the CDIs where the participants described no major difficulties in relation to the double negation; hence, no changes were made.

The final correction was in relation to three items regarding social events, which were combined with “like to a party” in a bracket. One participant could not relate to the question as she did not go to parties. She did not think a birthday was a party, so she suggested adding “birthday” to the bracket.

As a result of the changes in the fourth Danish translation of LIMB-Q Kids, two more CDIs were conducted to access the minor changes. Young patients were chosen as it was in this population that the difficulties were observed in the first CDIs, and no subsequent difficulties were observed.

### 3.5. Proofreading

The fifth Danish version of the LIMB-Q KIDS was independently proofread by two clinicians with expertise in PROM development. As no changes were found necessary, the final Danish version of the LIMB-Q Kids module was considered linguistically validated and culturally adapted to be used in Danish patients with lower limb deformities.

## 4. Discussion

The use of the combined guidelines from WHO and ISPOR has provided a useful and structured method for the TCA, resulting in a linguistically validated and culturally adapted Danish version of the LIMB-Q Kids. The expert panel and the CDIs have proven essential for the translation process by adding the clinician’s and the children’s perspectives.

### 4.1. Strengths and Limitations

The systematic use of WHO and ISPOR guidelines has been a major strength of this study and has ensured a high degree of validity [16]. Additionally, all CDIs were conducted by one person (first author), leading to consistency in the interview methodology, data logging, and data interpretation. To accommodate the diversity of the patient group, it was also decided that 10 CDIs should be conducted in the first round with a supplement of two interviews in the second round, compared to the ISPOR guidelines, where five to eight interviews are recommended [13].

The CDIs were conducted online as a result of the COVID-19 pandemic and to ensure effective logistical management for the patients. During the interviews, any hesitancy or indicators of confusion, for example, participants looking at their parents for help, were addressed and noted. The children were asked about their experience of being interviewed online. The children that participated felt safe being interviewed in their home environment. This was reflected in positive facial expressions and frequent eye contact between the interviewer and the children during the interview process. This is an important consideration in research, particularly when working with vulnerable populations such as children, as a safe and comfortable environment can help to ensure that participants are able to provide valid responses. An additional benefit was that it was easy for participants with reduced mobility to take part in the interviews. In general, online CDIs of children accompanied by a parent have shown to be a useful method, and they provide a safe environment for the children [17].

Another strength has been the close cooperation with the developers of LIMB-Q Kids (H.C, A.C) through every step of the translation process. If there has been a doubt about the connotations or the subtext of an English item, the cooperation made it possible to better evaluate the quality of Danish adaptions, which has been an indispensable asset.

In relation to the final Danish version of LIMB-Q Kids, two items based on the word “dislike” has proven to be a problem in the translation process (See Table 1). The CDIs did not show any comprehension problems, but although no changes were made to the items, the issue was noted by the developers of LIMB-Q Kids to see if the issue occurs in other translations.

A limitation of the current version of LIMB-Q Kids is the length, as the current form consists of 159 items. Meanwhile, LIMB-Q Kids consist of 11 independently functioning scales, meaning that users can pick and choose the scales that are clinically relevant for the patients, and use the scales independently without compromising the validity [2].

As a part of the validation process, LIMB-Q Kids is now being tested in an international field test study in multiple international sites, including one in Denmark, where the Danish translation is being used. A German TCA has also been conducted, and it is now being used in German-speaking sites [18]. Additional sites from Canada, UK, and the USA are also using the current version of LIMB-Q Kids as part of the international field test study. Arabic, Mexican Spanish, Hindi, Hebrew, Finnish and Portuguese translations are also in progress. The combined data from all sites will be used for psychometric validation. This will result in the scoring algorithms for the scales, and item reduction for poorly performing items according to the result of the Rasch analysis [2].

At a later stage, the LIMB-Q Kids could possibly be significantly shortened by utilizing adaptive computer testing. This is a method to shorten and tailor a PROM while maintaining validity, reliability, and accuracy through machine learning [19].

LIMB-Q Kids is specifically designed for children and adolescents. It is thus not developed to capture the HRQL of adults. Other PROMS exist for these groups of patients, which theoretically could be applied to the population of LIMB-Q Kids [20,21]. However, the development and conceptual framework are either not as rigorous, or the PROM is not designed for children. Comparing the results of LIMB-Q Kids to other PROMS, such as the LLRS-AIM, PROMIS, and PedsQL, will clarify the similarities and differences in more detail after the ongoing international field test [22,23,24].

### 4.2. Applicability and Development of the LIMB-Q KIDS Module

Today, many PROMs used in pediatric orthopedic studies do not have complete validation for the included population, which compromises the validity of the study in which it is used [1,10,25].

To our knowledge LIMB-Q Kids is the only validated PROM specifically designed to measure the HRQL of children with lower limb deformities [11]. LIMB-Q Kids will be the first PROM to measure 12 different concepts, focusing on the child’s physical function, symptoms, psychological function, social function, school function, leg-related distress, and appearance [11].

LIMB-Q Kids was developed as an international collaboration, and it is now being field tested internationally. This will be conducted by following international PROM developing guidelines, which ensure a high degree of validity and evidence of the finished product. As a result, LIMB-Q Kids will have a broad field of application internationally with the necessary validity [13].

In daily clinical practice, LIMB-Q Kids can be used as a tool in the course of treatment to evaluate the HRQL prior to an appointment with a healthcare professional. Data from LIMB-Q Kids can be used as a tool for shared decision-making, as it gives the clinician a unique insight into the patient’s health and state of mind. Afterward, LIMB-Q Kids can be implemented as a tool to assess the effectiveness of an intervention, such as a reconstruction procedure, amputation, or the use of a new prosthesis in terms of HRQL [1]. LIMB-Q Kids can potentially identify areas that are important to individual patients to improve the quality of care, and thereby make sure that the children’s happiness and well-being are addressed [12].

When the LIMB-Q Kids has been psychometrically validated, it can provide a high degree of sensitivity and validity in pediatric orthopedic studies [12]. Clinical trials evaluating different orthopedic interventions with classic objective measures in combination with data from LIMB-Q Kids make it possible to establish evidence for the superiority of one treatment over another. In addition to this, study data might find fields of interest identified by the PROM that can predict the expected gain in HRQL for different patient groups [2]. The international development and validation of LIMB-Q Kids make it possible to pool and compare data from various international sites, which can be crucial when dealing with rare orthopedic conditions and their treatments.

## 5. Conclusions

The Danish version of the LIMB-Q Kids questionnaire has been linguistically validated and culturally adapted according to the rigorous TCA process. Following international consensus guidelines during TCA is mandatory for the validity of the translated PROM.

Currently, an international field test of the LIMB-Q Kids is being conducted in 15 countries, aiming for a target of more than 500 participants. Rasch measurement theory will be applied. This process will lead to item reduction of the 159 items and 11 scales. After psychometric testing, the current validated advanced translation of LIMB-Q Kids will be available to be used in clinical practice and research to measure the impact of lower limb differences on the HRQL of children. Evaluating the HRQL will make it possible to follow the individual treatment courses. Moreover, it will be a valuable tool for assessing different types of surgical procedures with the patient’s perspective in focus.

## Figures and Tables

**Table 1:** Demographics of participants in the cognitive debriefing interviews. Male (M), female (F), hereditary multiple exostoses (HME), proximal femoral focal deficiency (PFFD), Taylor Spatial Frame (TSF), and TrueLok Hexapod System (TLHex).

Patient	Age (Years)	Gender (M/F)	Diagnosis	Type of Treatment	Stage of Treatment
	**First Round of Interviews**				
1	15	M	HME	Ring fixation tibia (TSF)	Ongoing
2	17	M	Post Traumatic Proximal Tibia	Ring fixation tibia (TLHex)	Ongoing
3	11	F	Genu Valgum	8-Plates	Planned
4	13	M	PFFD	Intramedullary femoral lengthening + 8-plates	Previous
5	15	M	Tall Stature	Hemiepiphysiodesis	Planned
6	13	F	Epiphysiolysis/Fracture	Intramedullary femoral lengthening	Ongoing
7	11	M	Slipped Caput Femoris Epiphysiolysis	Open repositioning and prophylactic contralateral pinning	Ongoing
8	10	F	Genu Valgum	8-plates	Finished
9	9	F	McCune Albright Syndrome, Left Femur Fracture in 2019	Gap-nail	Ongoing
10	8	F	Talus Fracture	Casting	Finished
	**Second Round of Interviews**				
11	10	M	Genu Valgum	Guided growth	Planned
12	8	F	Distal Tibia Epiphysiolysis	Guided growth, planned Acute correction with plate	Ongoing

## Data Availability

Data can be requested from the corresponding author at christopher.emil.jonsson@rsyd.dk.

## Supplementary Materials

The following supporting information can be downloaded at: https://www.mdpi.com/article/10.3390/children10071107/s1, Table S1: Overview of the translation process with Danish explanations.
