# Supplementary material for: Danish Linguistic Validation and Cultural Adaptation of the LIMB-Q Kids
Source: Children (Basel). 2023 Jun 25;10(7):1107. doi: 10.3390/children10071107 (PMC10377942; doi:10.3390/children10071107)
Supplement: Supplementary file 1 [file children-10-01107-s001.zip › children-2431697-supplementary.pdf]

Table S1. *Overview of the translation process with Danish explanations.*

Scale and Item number	Original item	Forward translation discussion	Backward translation Discussion	Expert Panel meeting Discussion	Cognitive debriefing interview Discussion
**1.2**<br>**2.2**<br>**3.2**<br>**4.2**<br>**5.2**<br>**6.2**<br>**7.2**<br>**8.2**<br>**9.2**	These questions ask about the leg you are seeing the doctor or nurse about.	No Comments	The Danish translation of 'you will be talking to the doctor or nurse about' were changed to a synonym closer to the original item. Changed from “snake med” to “tilses af”	It was decided to use another synonym for the translation of “seeing”. The Danish word “behandles” was chosen.	No Comments
**4.2**<br>**5.26**<br>**6.2**	My knee/Leg/ankle hurts when I stand for a long time.	No Comments	As the items were back translated to “My knee hurts when I stand up for a long time” The Danish word for up was removed from the Danish version.	No Comments	No Comments
**1.13**	…Walk as fast as you like?	No Comments	No Comments	Another synonym was chosen for the word “like” as it was concluded that the meaning of the word formerly chosen had a meaning closer to “Want”. It was changed from ”Vil” to ”har ”lyst til”	No Comments
**1.14**	…Walk as far as you like?	No Comments	No Comments		No Comments
**1.24**	…Run as fast as you like?	No Comments	No Comments		No Comments
**1.25**	…Run as far as you like?	No Comments	No Comments		No Comments
**2.12**<br>**4.13**<br>**5.10**<br>**6.9**	My leg/knee/ankle/foot is swollen or Puffy	No comments	No comments	It was decided to use only one synonym for “swollen” or “puffy” in the Danish translation, as it	No comments

				were found sufficient. The word "hævet" was chosen.	
**2.9**<br>**3.10**<br>**4.12**<br>**5.9**<br>**6.8**	My leg/Hip/Knee/Ankle/Foot feels weak (shaky)	No comments	No comments	Consensus was to use two synonyms to ensure the connotations of the word "shaky" in the Danish translation.<br>The Danish words "usikert" and "rystende" were chosen.	The majority of the participants thought the use of two synonyms made the item more easy to understand.
**1.5**	…sit cross legged on the floor?	No comments	No comments	No comments	Some patients could not understand the word "skræderstilling" which is a Danish word for sitting cross legged" So, it was decided to add it as a bracket combined with a more literal translation of the phrase. The patients thought the item were more easily understandable, after the change.
**1.12**	…walk on a bumpy road.	No comments	No comments	No comments	As three patients did not know the Danish word for "bumpy" it was decided to add a synonym in a bracket, to make it more easily understandable. The Danish word for potholes "hullet" was chosen, and the participants in the
**5.4**	My ankle hurts when I walk on a bumpy road.	No comments	No comments	No comments	

					second round of interviews had a better understanding of the item.
**7.3**	I dislike how my leg looks.	No comments	"Dislike" was back translated to "do not like", This did not result in a change as no better alternative were found.	A better Danish synonym for "dislike" was sought, but no good alternative was found.	The participants had no major problems, understanding the item, despite the double negation when choosing the response option "Never", hence no change was made.
**7.10**	I dislike how I look when I walk	No comments			
